# Concentration of circulating autoantibodies against HSP 60 is lowered through diving when compared to non-diving rats

**DOI:** 10.3402/mehd.v23i0.10677

**Published:** 2012-01-31

**Authors:** Marianne B. Havnes, Catrine Ahlén, Alf O. Brubakk, Ole-Jan Iversen

**Affiliations:** 1 Department of Circulation and Medical Imaging, Faculty of Medicine, Norwegian University of Science and Technology, Trondheim, Norway; 2 SINTEF Technology and Society, MT-Medical Microbiology, Trondheim, Norway; 3 Department of Laboratory Medicine, Children's and Women's Health, Faculty of Medicine, Trondheim, Norway

**Keywords:** saturation diving, infection, long-term effects, anti-HSP60, immunisation

## Abstract

**Objective:**

Skin and ear infections, primarily caused by *Pseudomonas aeruginosa* (*P. aeruginosa*), are recurrent problems for saturation divers, whereas infections caused by *P. aeruginosa* are seldom observed in healthy people outside saturation chambers. Cystic fibrosis (CF) patients suffer from pulmonary infections by *P. aeruginosa*, and it has been demonstrated that CF patients have high levels of autoantibodies against Heat shock protein 60 (HSP60) compared to controls, probably due to cross-reacting antibodies induced by *P. aeruginosa*. The present study investigated whether rats immunised with *P. aeruginosa* produced autoantibodies against their own HSP60 and whether diving influenced the level of circulating anti-HSP60 antibodies.

**Methods:**

A total of 24 rats were randomly assigned to one of three groups (‘immunised’, ‘dived’ and ‘immunised and dived’). The rats in group 1 and 3 were immunised with the bacteria *P. aeruginosa*, every other week. Groups 2 and 3 were exposed to simulated air dives to 400 kPa (4 ata) with 45 min bottom time, every week for 7 weeks. Immediately after surfacing, the rats were anaesthetised and blood was collected from the saphenous vein. The amount of anti-HSP60 rat antibodies in the serum was analysed by enzyme linked immunosorbent assay.

**Results:**

The immunised rats (group 1) showed a significant increase in the level of autoantibodies against HSP60, whereas no autoantibodies were detected in the dived rats (group 2). The rats both immunised and dived (group 3) show no significant increase in circulating autoantibodies against HSP60. A possible explanation may be that HSP60 is expressed during diving and that cross-reacting antibodies are bound.

Saturation diving is widely used in the North Sea for maintenance and inspection of off-shore sub-sea petroleum production systems. Exposure to hyperbaric environments is associated with a risk of developing decompression sickness (DCS), arterial gas embolism, neurological symptoms and pulmonary dysfunctions (1–3). Divers are usually compressed in their working and living environment for 12–24 days and are exposed to an ambient pressure of 0.6–2.1 MPa (50–200 msw). The hyperbaric working and living environment is warm and humid and so maintains a rich microbial flora (4).

Health problems, such as skin and ear infections, in divers working on the Norwegian Continental Shelf have been systematically registered since 1985 (4). Within the period from 1990 to 2010, four incidents of DCS and 201 incidents of outer ear infection were reported to the Petroleum Safety Authority Norway (5).

Skin and ear infections in saturation diving are usually caused by *Pseudomonas aeruginosa* (*P. aeruginosa*) (6). Such infections have been common since the beginning of saturation diving, and infectious outbreaks have caused costly breaks in operations (7, 8). The *P. aeruginosa* bacterium is commonly found all over the world, occurring in both fresh- and seawater, soil and on plants, and the species is noted for its metabolic versatility and its exceptional ability to adapt to and colonise various ecological niches.

Infections caused by *P. aeruginosa* are seldom observed in healthy people outside a saturation chamber, but the bacterium is well known as an opportunistic pathogen. Patients with cystic fibrosis (CF) suffer recurrently from pulmonary infections due to *P. aeruginosa* (9). Insulin-dependent diabetes is the most prevalent co-morbidity condition in CF (10), and it has been suggested that destruction of the insulin-producing beta-cells in the pancreas is caused by autoantibodies that act against heat shock protein 60 (HSP60) (11). HSP60 molecules are highly phylogenetically conserved with about 50% sequence homology between human HSP60s and those of *P. aeruginosa* (12). Thus, the presence of autoantibodies against HSP60 in patients with CF may be due to human antibodies cross-reacting in a process induced by the presence of bacterial HSP60.

Heat shock proteins are involved in folding and unfolding of other proteins (13) and are expressed in response to various stressors such as hyperoxia, hypoxia, heat, cold, exercise, some heavy metals and drugs, and many of these factors are involved in diving (14). HSP60, a member of this family, is highly expressed *in vitro* in endothelial cells. It is normally an intracellular protein, but in response to various stresses it is expressed on the surface (15). Binding of anti-HSP60 antibodies to HSP60 has been suggested to be present in the development of atherosclerosis (16). Furthermore, immunisation of mice with human sera containing high levels of anti-HSP60 induces atherosclerosis (17). They even found a marked induction of atherosclerotic lesions after a single injection of purified anti-HSP antibodies (17).

Hence, a relevant question is whether *P. aeruginosa* infections amongst saturation divers may induce production of autoantibodies that might cross-react and bind to human HSP60.

In the present study, we investigated whether rats immunised with *P. aeruginosa* produced autoantibodies against rat HSP60 and whether the autoantibody level was affected by diving.

## Material and methods

A total of 24 young female Sprague–Dawley albino rats (Scanbur, Denmark), weighing 0.262±0.013 kg, were used in the experiment. All animals used in the experiment were bought at the same time, from the same supplier and had equal amount of time for acclimatisation. All experimental procedures and the care of experimental animals conformed to the European Convention for the Protection of Vertebrate Animals Used for Experimental and Other Scientific Purposes, and the protocol was approved by the Norwegian Council for Animal Research.

Following 1 week of acclimatisation, the rats were randomly assigned to one of three groups, ‘Immunised’, ‘Dived’ and ‘Immunised and dived’ (*n*=8 for each group). There was no significant difference in weight between the groups.

One strain of *P. aeruginosa* (genotype E) isolated from an infected saturation diver was used in this study (4). The isolated bacterium was inactivated by 65°C for 30 min. Cultures were solved in sterile and filtered phosphate buffered saline (PBS) buffer (pH 7.2) and diluted to fit optical density (OD) of 600 nm To ensure that the vaccine is sterile, growth was examined by coating 100 µl of the vaccine on Blood Agar and incubating at 37°C for 2 days. The vaccine was preserved in aliquots at –80°C until vaccinations.

The rats in groups 1 and 3 were immunised with *P. aeruginosa* crude antigen, 0.2 ml every other week, from week 1 to week 9, in total five times. Two weeks after the first immunisation, groups 2 and 3 were exposed to simulated air dives. The rats only subjected to pressure exposure had injections of saline solution (0.9% NaCl, B. Braun, Melsungen, Germany), at the same time as the other ones had immunisations. The compressions were performed in a 20-L hyperbaric chamber with continuous air supply. Both dive groups were compressed (200 kPa/min) to 400 kPa (4 ata) with 45 min bottom time. The decompression rate was 50 kPa/min. The dive protocol in both groups was repeated every seventh day for 7 weeks, in total seven times.

Immediately after surfacing, the rats were anaesthetised with a subcutaneous injection of a mixture of Haldol 0.33 mg, Fentanyl 0.05 mg and Midazolan 0.5 mg at a dose of 2.5 ml/kg of body weight. Blood was collected from the saphenous vein. After blood sampling, the rats were moved to their housing facilities where they were allowed to recover.

Two weeks after the last test protocol was performed, the rats received a 1.2-ml subcutaneous injection of the same anaesthetic mixture as described previously and were sacrificed by heart puncture.

Serological analyses: Enzyme linked immunosorbent assay (ELISA) for detecting antibodies against rat HSP60.

Sera were analysed by indirect ELISA. Recombinant rat HSP60 (Stressgen, Victoria, Canada) was diluted at a ratio of 1:1,000 in coating buffer, which contained 1.59 g of Na_2_CO_3_, 2.93 g of NaHCO_3_ and 1 L of distilled water (pH 9.6), and 100 µl/well were incubated in microplates (Sterilin) overnight at room temperature. Plates were then washed three times with PBS containing 0.05% Tween 20 (PBS-T). Plates were pre-incubated at room temperature with 300 ml/well of blocking buffer, containing 1% skimmed milk powder (Molico, Nestlè) in PBS, for 30 min. After washing three times with PBS-T, wells were incubated in duplicate with rat serum in a dilution of 1:10 ml/well, in blocking buffer, for 2 h. Plates were washed three times with PBS-T and incubated with polyclonal rabbit anti-rat IgG/ HRP (DakoCytomation, Denmark), diluted at a ratio of 1:1,000 in blocking buffer, for 1 h at room temperature. After washing four times with PBS-T, 100 ml of freshly made Sigmafast OPD (containing 0.4 mg/ml o-phenylenediamine, 0.4 mg/ml urea hydrogen peroxide and 0.05 M phosphate-citrate in 20 ml distilled water, pH 5.0) (Sigma, USA) was added to each well. After 30 min, the reaction was stopped with 50 ml of 2 M H_2_SO_4_ and plates were read at 490 nm. OD values were derived from duplicate determinations.

### Statistics

The results are presented as mean±S.D. Mann–Whitney and the Wilcoxon signed-rank test were used to analyse the ELISA results. The level of statistical significance was set at *P*<0.05. All statistical analyses were performed using SPSS 14.0 (SPSS Inc., Chicago, Illinois, USA).

## Results

**Fig. 1:**
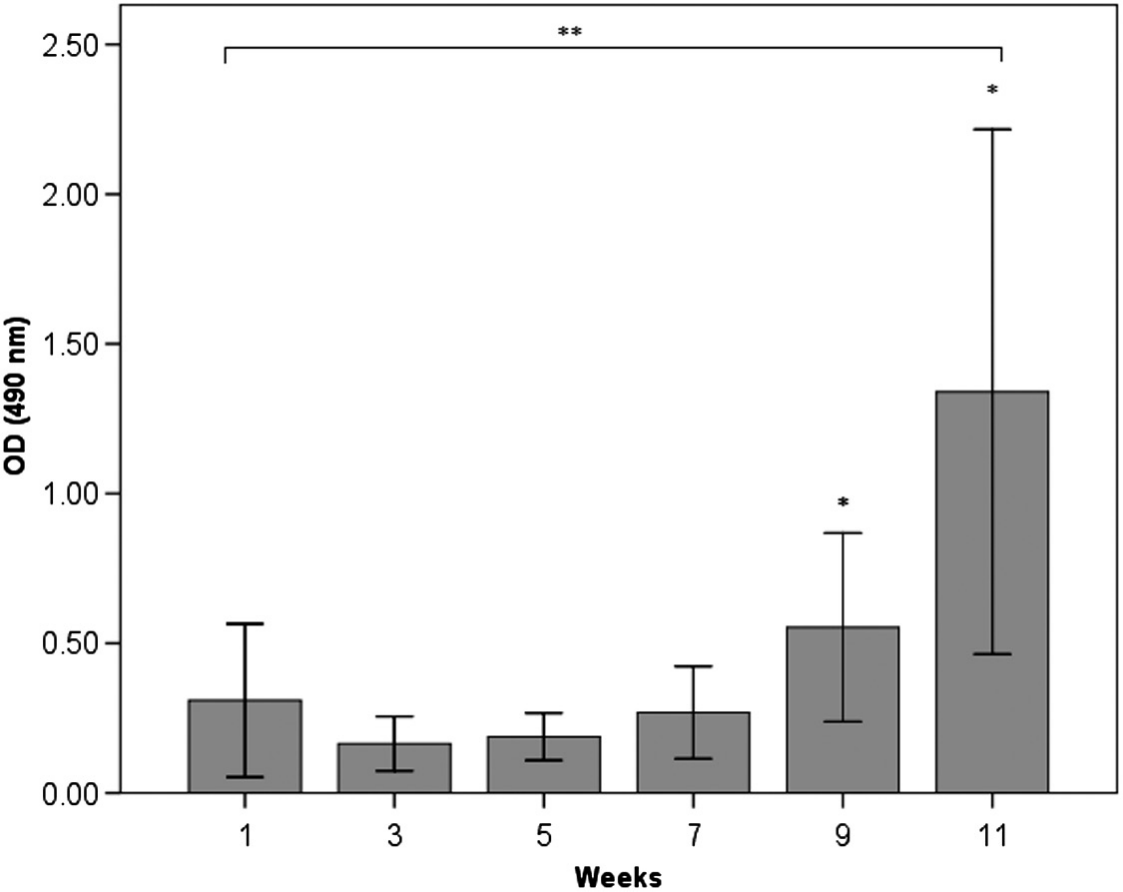
Longitudinal study of anti-HSP60 level in serum in the immunised group (group 1). Bars represent level of anti-HSP60 analysed by ELISA in blood samples taken every other week, before each immunisation. Notes: **There is a significant increase in anti-HSP60 level from week 1 to week 11 (p < 0.01). Bars represented by * are significantly different from same week in group 3 (Fig. 3). Results are presented as mean optical density (OD) values measured at 490 nm. The vertical lines represent the standard deviation.

**Fig. 2:**
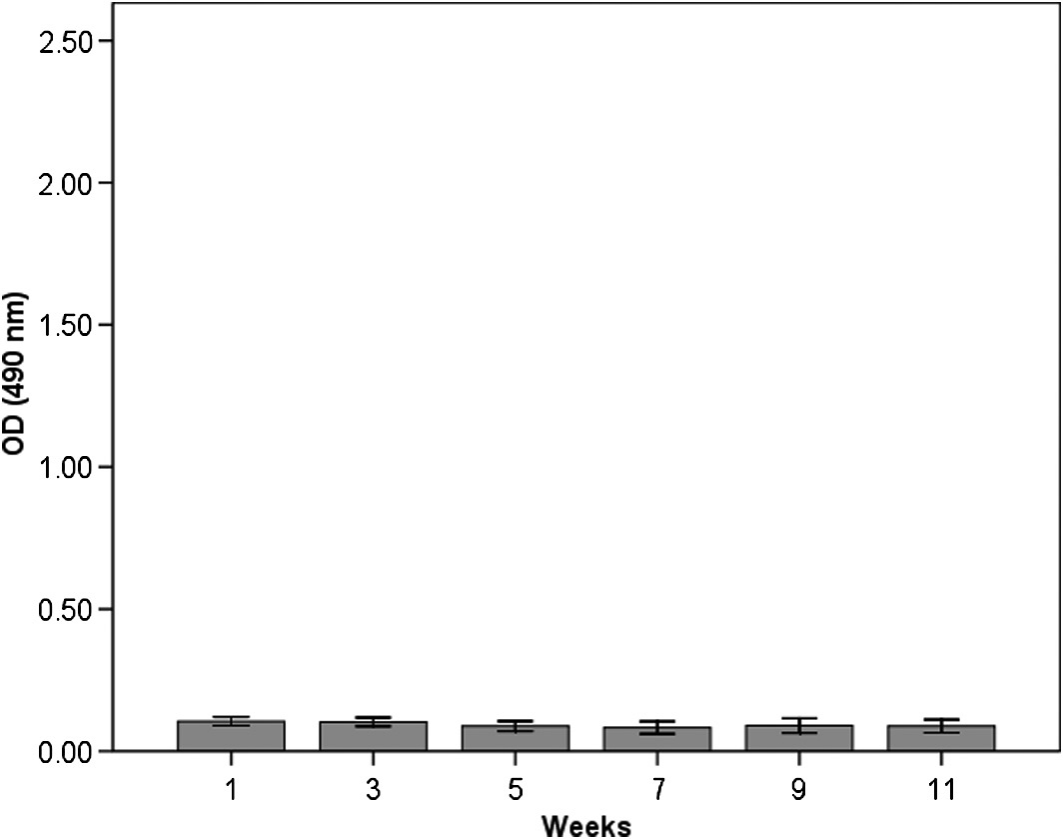
Longitudinal study of anti-HSP60 level in serum in the dived group (group 2). Notes: Bars represent level of anti-HSP60 analysed by ELISA in blood samples taken every other week/dive, immediately after surfacing/decompression. There is no change in circulating anti-HSP60. Results are presented as mean OD values measured at 490 nm. The vertical lines represent the standard deviation.

**Fig. 3:**
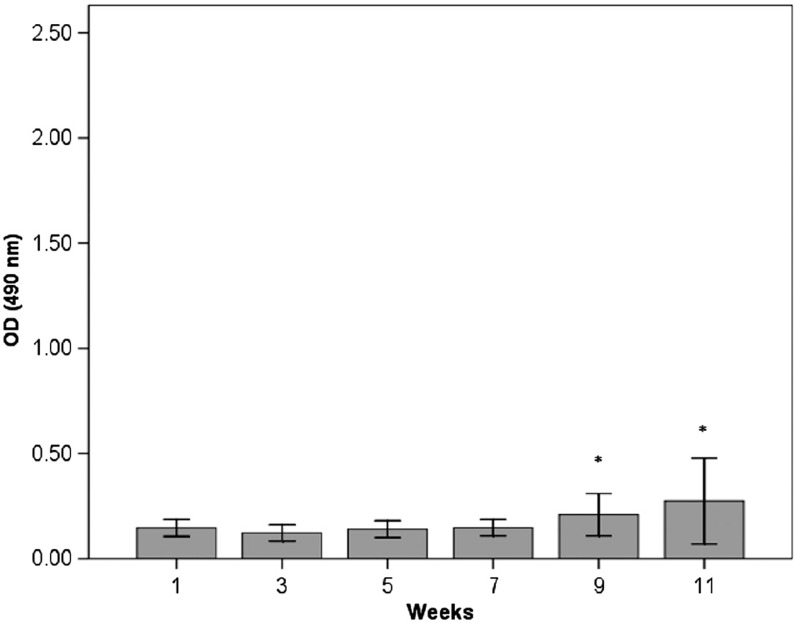
Longitudinal study of anti-HSP60 level in serum in the immunised and dived group (group 3). Notes: Bars represent level of anti-HSP60 analysed by ELISA in blood samples taken every other week/dive immediately after surfacing/decompression. In contrast to the immunised rats (group 1), there is no significant change in level of circulating anti-HSP60.Bars represented by * are significantly different from same week in group 1 (Fig. 1). Results are presented as mean OD values measured at 490 nm. The vertical lines represent the standard deviation.

The results from the ELISA measurements of anti-HSP60 antibodies in serum are shown in Figs 1, 2 and 3. In the immunised group (group 1), there was a significant increase in anti-HSP60 level from week 1 to week 11 (*p* ≤ 0.01) (Fig. 1). No significant increase in the concentration of free anti-HSP60 antibodies was detected in the diving group (group 2, Fig. 2) or in the immunised and dived group (group 3, Fig. 3). There are significantly higher levels of anti-HSP60 at week 9 (*p*=0.011) and 11 (*p*=0.006) in group 1, compared to group 3.

## Discussion

This study demonstrates that the level of circulating autoantibodies against HSP60 significantly increases over time in rats with repeated immunisations with *P. aeruginosa* (group 1). The dive rats (group 2) did not show any increase in the level of circulating antibodies during the experimental period, but more surprisingly, we were not able to detect circulating autoantibodies against HSP60 in immunised rats when the blood sample was taken immediately after diving (group 3).

The increased level of autoantibodies against HSP60 in the immunised rats in the current study is in accordance with the situation seen in patients infected by the bacteria *P. aeruginosa* (18). High levels of HSP60 autoantibodies are associated with an increased risk of coronary heart disease (19, 20) and have been associated with the development of type-1 diabetes in CF patients (11).

The animals only subjected to pressure did not show any increased level of circulating autoantibodies against HSP60. Hence, the pressure exposures did not themselves produce circulating autoantibodies.

When rats immunised with *P. aeruginosa* were exposed to dives, they were not observed to have the same rise in the level of circulating antibodies against HSP60 compared to the immunised, non-diving rats. One possible explanation for this may be that diving suppresses the immune response. An alternative explanation may be that HSP60 molecules are expressed on endothelial cells in such a way that the cross-reacting epitopes are exposed to the surface and, thus, enable binding between circulating anti-HSP antibodies and exposed HSP60 epitopes.

There is considerable research on cross-reacting autoantibodies in relation to development of atherosclerosis and in relation to several pathological reactions in CF patients. Bindings between anti-HSP60 antibodies to HSP60 have been demonstrated (16), and such bindings may in turn give rise to inflammatory reactions (11, 18–24).

We are not aware of any studies evaluating level of autoantibodies against HSP60 in saturation divers. Observations in the present study of higher autoantibody level after combining bacterial exposure and diving point in the direction of having cross-reacting autoantibodies. Hence, it seems likely that the exposure to *P. aeruginosa* in the diving environment and recurrent skin infections (6) may give rise to production of autoantibodies against HSP60.
